# Global dispersal and adaptive evolution of domestic cattle: a genomic perspective

**DOI:** 10.1007/s44154-023-00085-2

**Published:** 2023-04-18

**Authors:** Xiaoting Xia, Kaixing Qu, Yan Wang, Mikkel-Holger S. Sinding, Fuwen Wang, Quratulain Hanif, Zulfiqar Ahmed, Johannes A. Lenstra, Jianlin Han, Chuzhao Lei, Ningbo Chen

**Affiliations:** 1 Key Laboratory of Animal Genetics, Breeding and Reproduction of Shaanxi Province, College of Animal Science and Technology, Northwest A&F University, Yangling, 712100 China; 2 Academy of Science and Technology, Chuxiong Normal University, Chuxiong, 675000 China; 3 Qingdao Municipal Bureau of Agriculture and Rural Affairs, Qingdao, 266000 China; 4 Section for Computational and RNA Biology, Department of Biology, University of Copenhagen, Copenhagen, 1350 Denmark; 5 National Institute for Biotechnology and Genetic Engineering, Faisalabad, Pakistan; 6 Faculty of Veterinary and Animal Sciences, University of Poonch Rawalakot, Azad Jammu and Kashmir, 12350 Pakistan; 7 Faculty of Veterinary Medicine, Utrecht University, Utrecht, The Netherlands; 8 Livestock Genetic Program, International Livestock Research Institute (ILRI), Nairobi, 00100 Kenya; 9 CAAS-ILRI Joint Laboratory On Livestock and Forage Genetic Resources, Institute of Animal Science, Chinese Academy of Agricultural Sciences (CAAS), Beijing, 100193 China

**Keywords:** Cattle, Origin, Domestication, Migration route, Environmental adaptation, Selective pressure

## Abstract

Domestic cattle have spread across the globe and inhabit variable and unpredictable environments. They have been exposed to a plethora of selective pressures and have adapted to a variety of local ecological and management conditions, including UV exposure, diseases, and stall-feeding systems. These selective pressures have resulted in unique and important phenotypic and genetic differences among modern cattle breeds/populations. Ongoing efforts to sequence the genomes of local and commercial cattle breeds/populations, along with the growing availability of ancient bovid DNA data, have significantly advanced our understanding of the genomic architecture, recent evolution of complex traits, common diseases, and local adaptation in cattle. Here, we review the origin and spread of domestic cattle and illustrate the environmental adaptations of local cattle breeds/populations.

## Introduction

Domestic cattle are descended from the aurochs (*Bos primigenius*) (Ajmone-Marsan et al. 2010), which were widely distributed in Europe, Asia and northern Africa during the Holocene and went extinct in 1624 (Felius et al. 2014). Modern cattle were estimated have been domesticated ~ 10,000 years before present (BP) in Southwest Asia and ~ 8,000 years BP in South Asia (Larson et al. 2014; Pitt et al. 2019).

At present, approximately 1.5 billion cattle are kept on all inhabited continents, in a variety of climatic zones under diversified conditions (www.fao.org/faostat/en/). Domestic cattle are divided into humpless taurine cattle (*Bos taurus taurus*) and humped indicine/zebu cattle (*Bos taurus indicus*), local populations of which have undergone continuous admixture with other bovine species (Chen et al. 2018a; Chen and Lei 2021; Wu et al. 2018). Taurine cattle are largely confined to temperate and cold climates and are widely distributed in the Northern Hemisphere; some breeds are distributed in tropical Africa and America. Indicine cattle are found in southern Asia, Africa, northern Australia, the southern US, and Latin America (Utsunomiya et al. 2019; Zhang et al. 2020). Indicine cattle differ from taurine cattle in various ways: they exhibit a muscular fatty hump of variable size on their shoulders, a larger dewlap, drooping ears, and a tolerance of semiarid and tropical environments. Indicine cattle have a lower basal metabolic rate, water, and nutrient requirements. Moreover, they are generally more resistant to ticks and intestinal parasites than taurine cattle (Utsunomiya et al. 2019).

### Global dispersal of cattle

**Fig. 1:**
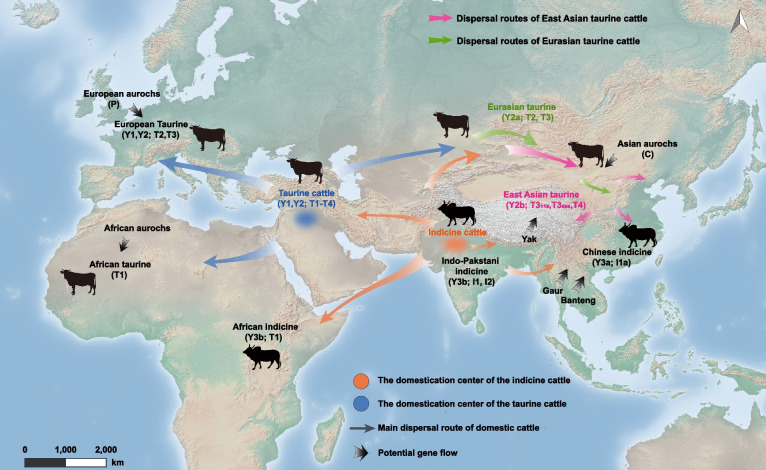
Domestication and main migration routes of *Bos taurus* and *Bos indicus*. Paternal (Y) and maternal (taurine, T; indicine, I; and aurochs, P and C) haplogroups are shown in parentheses, separated by a semicolon

Over the past 10,000 years, cattle domestication has been followed by several major migrations, leading to their presence on all inhabited continents (Felius et al. 2014). Environmental conditions of heat or cold, high altitudes or lowlands, and arid zones or humid tropical environments have contributed to the many adaptations of cattle and pronounced genomic diversity among breeds/populations (Fig. 1).

Taurine cattle were introduced from the Middle East into Africa 6,800 years ago (Felius et al. 2014) and probably influenced by local aurochs, which likely contributed to the ancestry of modern African taurine cattle (Pitt et al. 2019; Verdugo et al. 2019). The importation of male indicine cattle to Africa was initiated as early as 4,000 years ago and intensified at AD 700 (Ajmone-Marsan et al. 2010). At the end of the nineteenth century, cattle of the original taurine ancestry were largely replaced by indicine cattle, which were less vulnerable to devastating rinderpest epidemics (Felius et al. 2014). A major taurine-indicine admixture event was dated to approximately 750–1,050 years (approximately 150 generations) ago (Kim et al. 2020). This male-mediated indicine introgression into local taurine herds generated African indicine populations with a variable taurine/indicine genomic composition that are better adapted to dry climates. Over time, there was a stepwise transition from taurine to indicine diversity in Africa.

Approximately 4,200 years ago, climate change caused a male-mediated westward migration of indicine cattle from the Indus Valley to the Near East, which resulted in a change in genetic composition as cattle adapted to a dry climate (Verdugo et al. 2019). The eastward migration of taurine cattle reached the northern part of East Asia between 5,000 and 4,000 years ago, accompanied by the rapid adaptive evolution of ancestral taurine cattle to extremely low temperatures, as found in Siberia and the Qinghai-Tibetan Plateau (Felius et al. 2014). Around 3,000 years ago, indicine cattle migrated to Indochina and southern China. Following the contact of indicine cattle with early imports of taurine cattle, a north-to-south taurine-to-indicine cline was established at both the phenotypic and genomic levels in China (Gao et al. 2017). Intermediate taurine-indicine populations exhibited various combinations of taurine and indicine ancestries. The importation of indicine cattle to Southeast Asia likely began 1,500 years ago. Cattle were imported into North and South America from Europe and Africa since 1492 (Ajmone-Marsan et al. 2010; Felius et al. 2014). During their continuous dispersal in the tropical zones of Asia, Africa, and America, indicine cattle encountered southwestern and eastern Asian, African, and American taurine populations, respectively (Chen et al. 2018a; Utsunomiya et al. 2019), driving the emergence of several hybrid populations. Both southwestern Asia and central China are now characterized as typical taurine–indicine transition zones.

Molecular evidence of uniparental and autosomal markers has confirmed that taurine and indicine cattle are derived from two geographically separated and genetically differentiated aurochs progenitors from West and South Asia, respectively. Among modern cattle, there are seven major mitochondrial haplogroups (taurine T1, T2, T3, T4, and T5 as well as indicine I1 and I2) (Chen et al. 2010; Lenstra et al. 2014; Xia et al. 2019a); the rare mitochondrial haplogroups E, R, P, Q and C, supporting sporadic aurochs introgressions (Zhang et al. 2020; Xia et al. 2021; Cubric-Curik et al. 2022); five Y chromosome haplogroups (taurine Y1, Y2a, and Y2b as well as indicine Y3a and Y3b) (Xia et al. 2019b; Cao et al. 2019; Edwards et al. 2011; Pérez-Pardal et al. 2018); and at least eight major autosomal ancestral groups (Chen et al. 2018a, 2020) as follows: (1) African taurine cattle living in humid and sub-humid, tsetse fly-infested, tropical environments in West Africa (Gautier et al. 2009; Kim et al. 2017); (2) East Asian taurine cattle in Northeast Asia and the Qinghai-Tibetan Plateau, which are adapted to extremely cold and hypoxic environments, and some of them carry alleles arising from yak introgression (Chen et al. 2018a; Wu et al. 2018); (3) Eurasian taurine cattle in semiarid regions in Central Asia (Chen et al. 2021; Kantanen et al. 2009); (4) European taurine cattle inhabiting temperate climates that carry alleles arising through admixture with European aurochs and are the ancestors of most globalized industrial breeds (Achilli et al. 2008; Daetwyler et al. 2014; Park et al. 2015); (5) Indian-Pakistani indicine cattle in hot and semiarid regions (Chen et al. 2010); (6) African indicine cattle in semiarid East and Central Africa with a mixed ancestry of African taurine and South Asian indicine breeds (Bahbahani et al. 2017; Kim et al. 2017, 2020); (7) Diversified East Asian indicine cattle that inhabit hot-humid environments and carry alleles from other wild and/or domestic Asian bovine species (Chen et al. 2018a; Sinding et al. 2021); and (8) Indonesian breeds in hot-humid environments, which show a mix of indicine, banteng and/or Bali cattle ancestries (Mohamad et al. 2009; Sudrajad et al. 2020). For a more detailed and comprehensive classification of modern cattle, see Felius et al. (2011).

### Adaptation to a cold environment

**Fig. 2:**
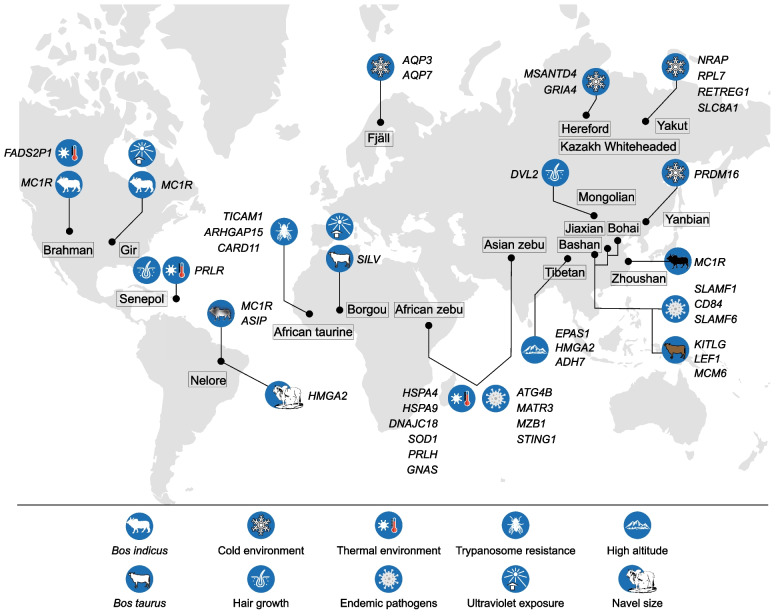
Examples of local adaptations in cattle breeds/populations, each labelled with the candidate gene or genes under selection

Cold climates are likely to affect the phenotypic characteristics and metabolism of cattle. Northern Fennoscandia and the Republic of Sakha, Russia, represent the northernmost regions inhabited by humans and are home to cattle breeds adapted to extremely cold environments (Weldenegodguad et al. 2018) (Fig. 2). For example, the Yakut cattle can be found above the Arctic Circle, and they have adapted to extremely cold winters (-50 °C). Recent genome-wide scans found that all Yakut cattle carry a breed-specific missense mutation in an evolutionarily conserved *NRAP* gene involved in heart function (Buggiotti et al. 2021) (Table 1). This change is shared by most hibernating mammals but absent from many mammalian species and other modern and ancient cattle breeds and bovine species. *NRAP* encodes the nebulin-related-anchoring protein enabling actin filament-binding activity and is abundantly expressed in striated and cardiac muscles involved in myofibrillar assembly and force transmission in the heart (Truszkowska et al. 2017). Thus, this young convergent NRAP amino acid change in Yakut cattle suggests that they may slow down their metabolism but enhance their heart function to supply blood during the winter periods (Buggiotti et al. 2021).

**Table 1:** Overview of known genes under local adaptation in cattle populations

Category	Breed/population	Method	Gene	Refs
Cold adaptability	Yakut cattle	RFMix, allele frequency	*NRAP*	(Buggiotti et al. 2021)
Cold adaptability	Russian native cattle	DCMS and hapFLK	*RETREG1*	(Yurchenko et al. 2018)
Cold adaptability	Hereford and Kazakh Whiteheaded cattle bred in Siberia	GWAS and DCMS	*MSANTD4*, *GRIA4*	(Igoshin et al. 2019)
Cold adaptability	Fjäll cattle	DCMS	*AQP3*, *AQP7*	(Ghoreishifar et al. 2020)
Cold adaptability	Yanbian and Mongolian cattle	*F*_ST_, Pi, Tajima's *D*	*PRDM16*	(Yan et al. 2022)
Navel size	Nellore cattle	GWAS	*HMGA2*	(Aguiar et al. 2018)
Heat adaptability	African and Asian zebus	LOTER, iHS, *F*_ST_, XP-EHH, XP-CLR, Hp	*HSPA4*, *HSPA9*, *DNAJC18*, *SOD1*	(Bahbahani et al. 2017; Kim et al. 2017, 2020)
Slick-hair phenotype and Thermoregulation	Senepol cattle	Association analysis	*PRLR*	(Littlejohn et al. 2014)
Thermoregulation	African zebu	XP-EHH	*PRLH*	(Kim et al. 2017)
Water reabsorption	African humped cattle	LOTER, iHS, *F*_ST_	*GNAS*	(Kim et al. 2020)
Heat adaptability	Brahman	*F*_ST_	*FADS2P1*	(Low et al. 2020)
Adaptation to endemic pathogens	African taurine cattle	PBS	*TICAM1*, *ARHGAP15*, *CARD11*	(Kim et al. 2020; Noyes et al. 2011)
Adaptation to endemic pathogens	African and Asian zebus	LOTER, iHS, *F*_ST_	*ATG4B*, *MATR3*, *MZB1*, *STING1*	(Kim et al. 2020)
Light coat color	Brahman, Nellore, and Gir	GWAS, di, Tajima’s *D*	*MC1R*, *ASIP*	(Mei et al. 2018; Trigo et al. 2021; Xu et al. 2015)
White to cream coat	Borgou cattle	iHS and Rsb	*SILV*	(Flori et al. 2014)
Dark brown coat	Chinese cattle	XtX statistics	*KITLG*, *LEF1*, *MCM6*	(Gao et al. 2017)
Black coat	Zhoushan cattle	*F*_ST_	*MC1R*	(Jiang et al. 2021)
Hypoxia adaptation	Tibetan cattle	*F*_ST_ and XP-EHH	*EPAS1*	(Wu et al. 2020)
Short stature	Tibetan cattle	*F*_ST_ and XP-EHH	*HMGA2*, *ADH7*	(Wu et al. 2020)
Adaptive immune responses	Jiaxian Red, Bashan, and Bohai Black cattle	*F*_ST_ and XP-EHH	*SLAMF1*, *CD84*, *SLAMF6*	(Ma et al. 2022; Sun et al. 2021; Xia et al. 2021)
Hair growth	Mongolian cattle	*F*_ST_ and θπ ratio	*DVL2*	(Mei et al. 2021)

Previous studies revealed several candidate genes that might be related to cold acclimation, including *RETREG1*, *RPL7*, and *SLC8A1*, in Yakut cattle of Russian (Weldenegodguad et al. 2018; Yurchenko et al. 2018). Among them, *RETREG1* (also known as *FAM134B*) is related to the impairment of pain and temperature sensation in humans (Kurth et al. 2009). *RPL7* encodes a component of 60S ribosomal subunit and showed a fourfold up-regulation in the skin of freeze tolerant frogs (Wu et al. 2018). *SLC8A1* encodes for solute carrier family 8 member A1 that is predominantly expressed in human heart and its mutations was found to be significantly associated with blood pressure rising during salt load (Liu et al. 2018). Another study on cold adaptation of Hereford and the Kazakh Whiteheaded cattle bred in Siberia for several decades identified a single candidate genomic region by both genome-wide association study (GWAS) and scan for selective sweeps, where *MSANTD4* and *GRIA4* were annotated. It was believed that both genes contribute to the cold-stress resistance phenotype due to their indirect involvement in the cold shock response (*MSANTD4*) and body thermoregulation (*GRIA4*) (Igoshin et al. 2019).

In northern Swedish cattle (Fjäll or Swedish mountain cattle) that also live in areas with a subarctic cold climate, signatures of positive selection were found near *AQP3* and *AQP7* (Ghoreishifar et al. 2020). *AQP3* and *AQP7* are mapped at the same chromosomal location as an aquaporin cluster and they encode the water channel protein aquaporin 3 and a member of the aquaporin family of water-selective membrane channels, respectively. AQP7 facilitates water, glycerol, and urea transport, and thus may play an important role in thermoregulation in the form of perspiration. Mongolian and Yanbian cattle in northern China with an annual average temperature of 2–6 °C harbor a substitution in *PRDM16* (p.P779L), which maintains brown adipocyte formation by boosting thermogenesis-related gene expression, indicating its vital role in cold tolerance (Yan et al. 2022).In northern Chinese cattle, *LCORL* has also been identified as a candidate gene for larger body size and greater height that may reflect local environmental effects (Lango Allen et al. 2010).

### Adaptation to tropical regions

Temperature increases lead to changes in forage quality and exacerbate livestock susceptibility to pests and diseases (Berman 2011). These factors cause physiological or behavioral changes in livestock, driving their adaptation to high-temperature environments. The body temperature is coordinated and controlled by the balance between metabolic heat production and loss (Bernabucci et al. 2014). Heat stress occurs when animals are exposed to high temperatures and cannot dissipate sufficient endogenous heat in time (Koch et al. 2019). Heat stress directly affects the food intake, growth, milk yield, and health conditions of domestic animals (e.g., heat shock), resulting in losses to production (Silva et al. 2021). Therefore, the study of thermal adaptation in cattle has become an important topic of research. Indicine cattle have several phenotypes that reflect adaptations to tropical environments, including their hump, large ears, and excess skin.

#### The hump, large ears and excess skin of indicine cattle

The hump is a muscular structure located above the withers on the dorsal region of the thoracic cage of indicine cattle; it is more prominent in males than females. The biological importance of the indicine hump remains unclear. Its abundant fat has inspired speculations regarding a role in energy storage in times of starvation, whereas the biomechanical relevance of the hump to the stabilization of the scapula suggests artificial selection for animal performance during draft (Utsunomiya et al. 2019). To date, no specific genomic region or gene has been implicated in the development of the hump.

Most indicine cattle carry large ears that are either spear tip-shaped or pendulous. Excess skin is usually present across the entire ventral midline, especially around the neck (throatlatch), chest (dewlap), and navel. A recent haplotype-based GWAS revealed that navel size was strongly associated with copy number variation at intron 3 of the high-mobility group AT-hook 2 gene (*HMGA2*) (Aguiar et al. 2018). The available evidence is not yet conclusive but suggests that structural variants (SVs) of *HMGA2* may explain the excess skin and ear morphology of indicine cattle, similar to findings in pigs (Li et al. 2012) and dogs (Boyko et al. 2010).

#### Skin morphology of indicine cattle

Cutaneous evaporation is the main avenue by which cattle dissipate heat, with the involvement of sweat glands and other skin components. A comparison of the skin morphology revealed denser, larger, and baggier sweat glands in indicine cattle with smaller capillary surface areas of hair follicles than those in taurine cattle, whereas the differences in skin morphology in their crossbreds correlated with the proportion of taurine ancestry (Jian et al. 2014). Likely the combination of large ears and excess skin with high density and effective sweat glands provides a smart, adaptive tolerance of indicine cattle to heat stress.

#### Positively selected genes associated with thermotolerance

Heat tolerance is a well-known characteristic of indicine cattle (Hansen 2004) and a prerequisite for indicine survival in hot climates. Genomic selection studies on African and Asian indicine cattle have identified a large number of candidate genes associated with heat tolerance. A subset of these genes (such as *HSPA4*, *HSPA9*, *DNAJC18*, and *SOD1*) are under selection (Bahbahani et al. 2017; Kim et al. 2017, 2020).

The prolactin signaling pathway not only is involved in lactation but also affects the hair morphology and thermoregulatory phenotype of cattle. Mutations in the 11th exon of the prolactin receptor (*PRLR*) have been shown to have a major effect on the slick-hair phenotype of cattle (Flórez Murillo et al. 2021; Littlejohn et al. 2014; Porto-Neto et al. 2018). The first slick mutation was found in the Senepol cattle, a tropically adapted breed of mostly European cattle descent, resulting in truncation of the C-terminal region of the protein involved in STAT5 activation during prolactin signaling (Littlejohn et al. 2014). Cattle with an extremely short and slick-hair coat show strong thermotolerance to withstand hot weather (Dikmen et al. 2014; Olson et al. 2003). Interestingly, the analysis of African cattle genomes also revealed a significant selective signal in prolactin releasing hormone gene *PRLH*, of which a missense mutation (p. Arg76His) in its exon 2 was highly conserved in African indicine cattle (73%) but absent in commercial taurine breeds, indicating its selective advantage by regulating prolactin expression relevant to thermotolerance in African indicine cattle (Kim et al. 2017).

Heat stress increases sympathetic nerve activity in kidneys, muscle, and skin (Rowell 1990). A genomic region with the access of indicine ancestry (92.44%) was found on *Bos taurus* chromosome (BTA) 13:57.15–57.65 Mb (Bahbahani et al. 2017; Kim et al. 2017, 2020), where GNAS complex locus gene is annotated. This gene is related to water reabsorption through mediating the antidiuretic hormone arginine vasopressin in aquaporin-2 water channels and subsequently contributing to the water conservation pathway of kidney (Boone and Deen 2008). This finding suggested that this specific indicine haplotype contributes to the local adaptation of African humped cattle to arid climate (Kim et al. 2020).

#### Adaptation to endemic pathogens

Pathogenic burden is an important driver of adaptation to tropical environments in cattle. Bovine trypanosomiasis, a vector-borne parasitic infection caused by *Trypanosoma* spp*.*, has long been a constraint on cattle farming in sub-Saharan Africa. Some African taurine cattle such as N'Dama, can withstand infection by *Trypanosoma congolense*. Genetic analysis has revealed selective sweeps at *TICAM1* and *ARHGAP15* loci in African taurine cattle, which were linked to previously identified quantitative trait loci (Noyes et al. 2011). Another selection signature in African taurine cattle is located in the upstream of *CARD11* (Kim et al. 2020), which is essential for the signaling of T and B cells in the innate and adaptive immune systems (Hara et al. 2003; Pomerantz et al. 2002). Furthermore, *CARD11* has been found to be differentially expressed between the trypanotolerant (N’Dama) and the trypanosusceptible (Boran) breeds (Noyes et al. 2011).

Candidate selective loci on BTA7 (*MATR3*, *MZB1*, and *STING1*) and BTA3 (*ATG4B*) with the excess of indicine ancestry were identified in both African humped as well as Asian and American-Australian indicine cattle, suggesting their possible contribution to genetic resistance to ticks and tick-borne diseases such as East Coast fever (Kim et al. 2020). STING1 regulates the production of intracellular DNA-mediated type I interferon and is thus essential for host defense against DNA pathogens (Ishikawa et al. 2009).

### Adaptation to ultraviolet exposure

Coat color variation may contribute to the adaptation of cattle to tropical/subtropical or high-latitude environments. Many indicine breeds, such as Nellore, Tharparkar, Bhagnari, Dajal, Hariana, and Guzerat, have light colors that can reflect a large proportion of incident solar radiation (Hansen 2004). The mixture of short, thick, densely arranged white/gray and dark hairs that cover the black skin of indicine cattle provides reflectance at short light wavelengths. Other studies have shown positive selection for the melanocortin 1 receptor (*MC1R*) gene in indicine cattle (Brahman, Nellore, and Gir), implying that their light coat color has played an important role in adaptation to tropical environments (Mei et al. 2018; Xu et al. 2015). The uniform white to cream coat of Borgou cattle is likely the result of artificial selection on the candidate gene *SILV* (Flori et al. 2014). This gene encodes a type I integral membrane protein in the premelanosome matrix (*PMEL17*), which is essential for melanosome development and is responsible for lightening or diluting the base color defined by the *MC1R* in some cattle breeds (Kühn and Weikard 2007; Schmutz and Dreger 2013).

The Nellore breed has been strongly selected for white coat, but bulls of this breed generally exhibit darker hair, ranging from light gray to black, on the head, neck, hump, and knees. GWAS has shown that this darkness is associated with a deletion of 1,155 bp followed by a small SINE-1 insertion (more than 150 bp) between the 1B and 1C noncoding exons of *ASIP* (Trigo et al. 2021). *ASIP* plays a crucial role in decreasing eumelanin and increasing pheomelanin production by blocking *MC1R* (Barsh et al. 2000; Cieslak et al. 2011). Thus, this SV of *ASIP* may cause darker coat pigmentation on specific parts of the body by decreasing the expression of *ASIP* and consequently increasing the production of eumelanin.

The common denotation of yellow cattle for all indigenous Chinese cattle refers to its predominant light to dark brown color. Using a whole-genome scan for genetic differentiation and association analyses with both environmental and morphological covariables, several coat color and pigmentation genes (*KITLG*, *LEF1*, and *MCM6*) were identified in Chinese cattle and considered to be involved in UV protection (Gao et al. 2017). Black Angus and Wagyu are typical black-coat taurine cattle breeds, while Zhoushan is an endangered black-coat indicine breed in southern China. The identification of a shared genomic region between Zhoushan and Angus cattle shows that the dark coat color of Zhoushan cattle may be related to the p.F195L mutation in MC1R (Jiang et al. 2021).

Although many genetic variants associated with cattle coat color have been identified (Mei et al. 2018; Trigo et al. 2021), little is known about the genetic basis of light coat color in indicine cattle in Asia and Africa. Indeed, coat color in Asian and African indicine cattle exhibits high variability, and the genetic basis of adaptations involving coat color requires further study.

### Adaptation to high altitude

High altitudes (> 2,500 m above sea level) in regions, such as the Qinghai-Tibetan Plateau, the Rocky Mountains of the USA, and the Simien Mountains Plateau of Ethiopia, can cause hypoxia due to an insufficient supply of oxygen to vital organs. However, cattle populations have thrived in these regions for thousands of years as a result of various physiological adaptations to hypoxic environments (Newman et al. 2015; Wang et al. 2021; Wu et al. 2020).

*EPAS1* encodes a subunit of the HIF transcription factor and is a key gene for hypoxia adaptation in Tibetans (Beall et al. 2010; Simonson et al. 2010; Yi et al. 2010). Recently, this gene was found to have evolved under positive selection in Tibetan cattle (top 5% ranking), corroborating the previously reported convergence of genetic adaptation to high altitude in dogs and humans (Wu et al. 2020). Some highly differentiated nonsynonymous SNPs were found in *EPAS1* of Tibetan cattle, which likely contribute to their local adaptation (Wu et al. 2020).

Similarly, a strong high association of double variants in the oxygen degradation domain of *EPAS1* has been found in Angus cattle in the Rocky Mountains, where they suffer from high-altitude pulmonary hypertension (HAPH) (Newman et al. 2015). These variants likely represent gain-of-function mutations that are prevalent in Angus cattle found at low altitude, but may be pathogenic under hypoxic conditions at high altitudes (Newman et al. 2015).

Short stature in adult Tibetan cattle, which have an average height of less than 110 cm, is another distinctive phenotype thought to contribute to their adaptation to the Qinghai-Tibetan Plateau. *HMGA2* has been identified as a candidate gene associated with the high-altitude adaptation of humans and domestic animals (Kader et al. 2015; Weedon et al. 2008, 2007). This gene was found to be positively selected for in Tibetan cattle (Wu et al. 2020). Notably, a nonsynonymous SNP (p.A64P in *HMGA2*) has a higher frequency in Tibetan cattle than in other cattle populations. *ADH7*, another gene that is associated with short stature in Tibetan cattle (Wu et al. 2020), has undergone positive selection in Tibetan cattle and is correlated with human weight and the body mass index (Weedon et al. 2008).

### Potentially selected genes related to environmental adaptation in other local cattle populations

Adaptation to local climate conditions is also being studied in other cattle populations. Chinese cattle still serve as a major labor force in agricultural production and are well known for their endurance and adaptive ability (Randhawa et al. 2016).

Genomic selection signatures of cattle breed in northern (Mongolian cattle) and southern China (Minnan cattle) identified several adaptive genes related to local environmental challenges, such as *DVL2*, *HSPA4*, and *CDHR4* (Mei et al. 2021). *DVL2* plays an important role in limiting hair growth and links to the hair follicle cycle (Gutiérrez-Gil et al. 2017). The missense mutations in *DVL2* show significant north–south population stratification and might impact both fur growth in different cattle breeds and adaptation to different climates (Mei et al. 2021). A region on BTA3 that includes the signaling lymphocytic activation molecule family (SLAMF) genes *SLAMF1*, *CD84*, and *SLAMF6* might be associated with high disease resistance in native Chinese cattle breeds, such as Jiaxian Red, Bashan, and Bohai Black cattle (Ma et al. 2022; Sun et al. 2021; Xia et al. 2021). SLAMF receptors are involved in the regulation and interconnection of innate and adaptive immune responses.

### Adaptive introgression

#### Yak introgression into Tibetan taurine cattle

Yak (*Bos grunniens*) are thought to have inhabited the Tibetan Plateau for millions of years and have high altitude adaptations, such as enlarged lungs and hearts. In contrast, domestic taurine cattle were introduced to the Tibetan Plateau by humans only a few thousand years ago (Chen et al. 2015). Taurine cattle that were not adapted to the Tibetan Plateau suffered from severe pulmonary hypertension in the early period (Will et al. 1975). Yak introgression into Tibetan cattle genomes partially facilitated cattle adaptation to high altitude (Chen et al. 2018a; Wu et al. 2018). Several adaptive introgressed genes have been identified that are related to the response-to-hypoxia pathway (for example, *COPS5*, *IL1A*, *IL1B*, *MMP3*, *EGLN1*, *EGLN2*, *HIF3*α, *RYR2*, and *SDHD*).

#### Introgression of banteng-like sequences into East Asian indicine cattle

East Asian indicine cattle are unique in caring for significant exotic ancestry, which is related to and can be modeled as gene flow from banteng (*Bos javanicus*) (Chen et al. 2018a, 2018b). However, while the banteng provides a good genetic match, it is not the precise source of East Asian indicine cattle (Sinding et al. 2021), indicating that more wild *Bos* diversity needs to be sequenced to fully explain their evolution. Nevertheless, using the Javan banteng as a reference is informative, and it has been inferred that between 2.38 and 3.84% of the southeastern Chinese indicine genome is of banteng ancestry (Chen et al. 2018a). Analysis of nonsynonymous substitutions in the introgression region led to the identification of the genes, such as *T2R12*, *TAS2R9*, and *TAS2R6.* These are homologues of bitter taste receptors in humans and giant pandas (Meyerhof et al. 2010; Zhao et al. 2013) may serve similar functions in East Asian indicine cattle. Although a clear correlation between bitterness and toxicity has not been established, it is generally believed that this taste ability prevents mammals from intoxication by avoiding ingestion of potentially harmful food constituents (Meyerhof et al. 2010). In addition, several introgressed genes conducive to the local adaptation of East Asian indicine cattle to the hot and humid tropical climate have been detected (Chen et al. 2018a). For example, several heat-shock protein (HSP) genes, including *HSPA1A*, *HSPB8*, *HSPA8*, *HSPA4*, *HSPB2* and *HSF2*, are involved in key cellular defense mechanisms during exposure to hot environments. Genes related to hair cell differentiation and blood circulation, such as *ATOH*, *GNA14*, *VPS13* and *KIF2B*, also play important roles in temperature adaptation (Chen et al. 2018a).

#### Introgression of banteng segmenst into Indonesian indicine cattle

Indonesia is home to Bali cattle, a local domesticated version of the Javan banteng, either or both of which are admixed into local Indonesian indicine breeds such as Galekan and Madura (Mohamad et al. 2009; Sudrajad et al. 2020), to the extent that this admixture likely has adaptive implications, hopefully future research will clarify this interesting question.

### New approaches to exploring genetic adaptation

#### Unearthing cattle adaptation with ancient DNA (aDNA)

aDNA sequencing provides a historical record of genomic variation. It provides new possibilities for identifying introgressions of wild stock and for studying domestication at the genomic level. Comparisons of early domestic cattle genomes in the Fertile Crescent to the genomes of their aurochs progenitors revealed diverse origins with separate introgressions of wild stock, such as British and Moroccan aurochs introgression into Neolithic Balkans and Levantine cattle, indicating that genetic exchange among early domestic groups and wild progenitors widely contributed to the development of domestic cattle (Verdugo et al. 2019). A genome comparison of British aurochs with modern European cattle revealed a number of genes associated with neurobiology, growth, metabolism, and immunobiology that show evidence of having undergone positive selection within the time since cattle domestication ~ 10,000 years ago. The analysis further showed significant introgression from the British aurochs into modern British and Irish cattle (Park et al. 2015). The environmental differences between Europe and Southwest Asia where cattle originated are notable. It is likely that the adaptation of European Neolithic cattle to a cold and wet Europe involved local aurochs introgression. Mitochondrial data and archaeological evidence revealed that East Asian aurochs, which belonged to the C haplogroup, were distributed in northern China during the Holocene and overlapped with early domestic cattle for millennia, possibly also contributing to the formation of modern East Asian cattle, a possibility that future research will hopefully clarify (Brunson et al. 2016; Cai et al. 2018; Zhang et al. 2013). Together, these findings suggest that additional aurochs populations have contributed to local cattle, which calls for future research into aurochs genomics. Furthermore, Verdugo et al. (2019) identified rapid and widespread introgression of indicine cattle in southwest and central Asia ~ 4,200 years ago, which was likely associated with climate change that led to the preferential use of arid-adapted indicine bulls for breeding. aDNA therefore has incredible potential to reveal not only aurochs evolution and the origin of cattle but also the transitions and breeding transformation of cattle, and much history has yet to be explored.

#### Adaptive potential arising from structural variation (SV)

Compared to smaller variants, SVs of at least 50 base pairs (bp) in length often have more extreme consequences (Chiang et al. 2017) and thus may have made substantial contributions to cattle adaptive evolution. For example, an additional copy of *FADS2P1* has been under positive selection in Brahman cattle (Low et al. 2020). It is a pleiotropic gene involved in the biosynthesis of unsaturated fatty acids, lipid homeostasis, inflammatory response, and promotion of myocyte growth and cell signaling. Its additional copy in indicine cattle may very well modulate water permeability and heat loss from skin by regulating the composition of fatty acids in the cell membranes (Low et al. 2020). However, SVs are extremely challenging to detect via short-read sequencing technologies. An accurate identification and characterization of SVs on the genome requires long-read sequencing technologies, novel computational approaches (Ho et al. 2020), and the availability of pangenome that represents the genome variations of a wide panel of cattle (Crysnanto et al. 2021; Leonard et al. 2022; Talenti et al. 2022; Zhou et al. 2022; Gong et al. 2022).

#### Integration of phenotypic, genetic, and functional data into adaptation studies

In the study of cattle environmental adaptability, phenotypic traits are easily distinguished. “Omics”-based analysis, such as transcriptomics and epigenomics, facilitates the study of functional variations that affect nonobvious or intermediate phenotypes. Furthermore, the integration of genome-wide selection scans with GWAS helps map putative regions of functional influence. This is particularly important in studies of indigenous populations, in which obtaining large sample sizes is challenging. Last, histological analyses or functional experiments can directly establish the links between candidate adaptive variants and phenotypes. For example, analyses combining GWAS and functional validation of mutations suggest that *PRL* and *PRLR* affect thermoregulatory and hair-morphology phenotypes in cattle (Flórez Murillo et al. 2021; Littlejohn et al. 2014; Porto-Neto et al. 2018).

## Conclusions and perspectives

After domestication, cattle spread around the world with human migrations. Selection pressures in response to regional conditions have affected global cattle genome diversity. The collection of global genome-wide population genetic data has led to the discovery of several examples of local adaptations to diverse environments. Further progress will depend on research in several areas as follows:

- High-coverage long-read whole-genome sequencing of local diverse populations can be used to construct pangenomes, which will include a large part of the global repertoire of SNPs and SVs.
- Whole-genome sequencing of aurochs and ancient cattle will enable hypotheses of the genetic consequences of recent artificial and natural selection in domestic cattle to be tested.
- Extending current GWAS-based methods to detect polygenic adaptations in combination with climate and environmental data is an important future direction.
- Detailed phenotyping will be stimulated by artificial intelligence approaches (Liang et al. 2021) and/or on integrative omics datasets (Peng et al. 2020).

These lines of progress are expected to illuminate the mode and tempo of local adaptation as large numbers of cattle settled in all inhabited continents.

## Data Availability

Not applicable.

## Abbreviations

BP: Before present
GWAS: Genome-wide association study
BTA: *Bos taurus* chromosome
aDNA: Ancient DNA
SV: Structural variant
Bp: Base pairs
Mb: Megabases
DCMS: Decorrelated composite of multiple signals
Pi: Nucleotide diversity
*F*_ST_: Fixation index
iHS: Integrated haplotype score
*Hp*: Pooled heterozygosity
XP-EHH: Cross-population extended haplotype homozygosity
XP-CLR: Cross-population composite likelihood ratio
PBS: Population branch statistics
XtX statistics: Population differentiation
θπ ratio: Genetic diversity ratio
hapFLK: Haplotype-based statistic
